# Targeting YY1-DR5 Axis by Pyripyropene O as a Novel Therapeutic Strategy Against Prostate Cancer: Molecular Mechanisms and In Vivo Zebrafish Validation

**DOI:** 10.3390/md23050214

**Published:** 2025-05-19

**Authors:** Wenxuan Fang, Ying Chen, Mingyi Nie, Xuefeng Zhou, Yonghong Liu, Huaming Tao, Bin Yang, Xueni Wang

**Affiliations:** 1 Guangxi Engineering Research Center for High-Value Utilization of Guangxi-Produced Authentic Medicinal Herbs, Institute of Traditional Chinese and Zhuang-Yao Ethnic Medicine, Guangxi University of Chinese Medicine, Nanning 530200, China; fangwenxuan2021@126.com (W.F.);; 2 Guangxi Key Laboratory of Marine Drugs, Institute of Marine Drugs, Guangxi University of Chinese Medicine, Nanning 530200, China; 3 Guangdong Key Laboratory of Marine Materia Medica/State Key Laboratory of Tropical Oceanography, South China Sea Institute of Oceanology, Chinese Academy of Sciences, Guangzhou 510301, Chinaxfzhou@scsio.ac.cn (X.Z.); 4 School of Traditional Chinese Medicine, Southern Medical University, Guangzhou 510515, China

**Keywords:** pyripyropene O, prostate cancer, apoptosis, Ying Yang 1, death receptor 5

## Abstract

Background: Induction of apoptosis is an important strategy for the treatment of prostate cancer. DR5 is a member of the death receptor superfamily and targeting DR5 is an effective way to induce apoptosis. Pyripyropene O is a natural compound isolated from the marine fungus *Aspergillus fumigatus* SCSIO 41220. We found it has anti-prostate cancer potential by inducing apoptosis; Methods: The effects of pyripyropene O on the viability, proliferation, cell cycle, apoptosis and migration of prostate cancer cells were investigated by MTT assay, plate clone formation assay, 3D cell sphere assay, flow cytometry and real-time cell analysis. Transmission electron microscopy was used to observe the changes in the internal structure of prostate cancer cells after treatment with pyripyropene O. After determining the mode of cell death, the mechanism of action of pyripyropene O on prostate cancer was further investigated using apoptotic protein microarray, western blot, qPCR, molecular docking, cellular immunofluorescence staining and cellular thermal shift assay. After explaining the mechanism of action of pyriproxyfen O, the in vivo absorption, distribution, metabolism, excretion and potential toxicity of pyriproxyfen O were investigated using ADMETLab 2.0 software. Finally, a zebrafish xenograft tumour model was developed to evaluate the anti-prostate cancer effects of pyriproxyfen O in vivo; Results: The experimental results at the cellular level showed that pyripyropene O inhibited the survival, proliferation and migration of prostate cancer cells, and also showed that pyripyropene O blocked the prostate cancer cell cycle at the G2/M phase and induced apoptosis. At the molecular level, pyripyropene O binds to the transcription factor YY1, promotes YY1 nuclear translocation, regulates the transcription level of DR5, a target gene of YY1, and upregulates the expression of DR5 mRNA and protein. The in vivo results showed that pyripyropene O effectively inhibited the development of prostate cancer in zebrafish; Conclusions: Pyripyropene O has a clear anti-prostate cancer effect at both cellular and animal levels, inhibiting the survival and proliferation of prostate cancer cells by binding to the transcription factor YY1 to activate the expression of DR5 to promote apoptosis, thus exerting an inhibitory effect on prostate cancer.

## 1. Introduction

Prostate cancer is one of the most common cancers of the reproductive system in middle-aged and older men [1]. With innovations in technology, treatments for prostate cancer have come a long way, including watchful waiting/surveillance, hormone therapy, surgical removal of the primary tumor, curative or palliative brachytherapy, cryotherapy, and curative or palliative chemotherapy [2]. However, surgical interventions are associated with a risk of urinary incontinence, erectile dysfunction, and urethral stenosis. Radiotherapy may lead to complications such as radiation cystitis, proctitis, and sexual dysfunction. Endocrine therapy can result in breast tissue development and osteoporosis, while chemotherapy is known to cause bone marrow suppression and gastrointestinal adverse effects [3]. In short, current therapies have their limitations. Therefore, exploring novel and efficient therapeutic targets for prostate cancer remains a difficult and hot research topic.

Compared with traditional anti-cancer methods, targeted therapy has gradually become an important means of cancer treatment due to its ability to selectively kill tumor cells without killing or minimally damaging normal cells, as well as its smaller adverse effects and better patient tolerance [4]. For prostate cancer, targeted therapy significantly improves efficacy through precise intervention in the core molecular mechanism of prostate cancer [5]. Currently, the main targets of anti-prostate cancer targeted drugs include androgen receptor (AR) signaling pathway inhibitors (e.g., abiraterone, enzalutamide), which play a therapeutic role by blocking testosterone synthesis or competitively inhibiting androgen binding [6,7]. PARP inhibitors (olaparib, etc.) targeting DNA repair defects (e.g., BRCA1/2 mutations) can selectively kill tumor cells using a “synthetic lethal” effect [8]. Aberrant activation of inhibitors of the PI3K/AKT/mTOR pathway has been strongly implicated in the progression of desmoplasia-resistant prostate cancer [9]. Radionuclide therapies targeting prostate-specific membrane antigen (PSMA) (e.g., 177Lu-PSMA-617) achieve tumor cell killing through precision radiation [10]. These emerging targeted anti-prostate cancer drugs bring hope to patients in their fight against cancer.

In fact, 70% of the structural scaffolds identified in marine natural products (MNP) are unique to marine organisms and have greater chemical novelty than terrestrial scaffolds [11]. Currently, there are 14 marine-derived drugs on the market, 9 of which are used to treat cancer [12]. Unfortunately, there are no marine drugs on the market that specifically target prostate cancer, but many compounds have been reported in preclinical and early Phase I/II clinical trials [12,13]. To date, no marine natural products have been identified that target the YY1/DR5 axis against prostate cancer. Our group has previously reported a variety of marine natural products with anti-prostate cancer properties, and no marine natural products targeting the YY1/DR5 signaling axis have been identified [14,15,16,17,18,19,20,21]. Targeting the YY1/DR5 axis against prostate cancer is a novel strategy.

Pyripyropene O (PyrO) in this study was derived from the marine fungus *Aspergillus fumigatus* SCSIO 41220. It was previously reported that pyripyropene O was first found in the ethyl acetate extract of the canned fermentation broth of *Aspergillus fumigatus* FO-1289-2501 in 1996, and it was found that pyripyropene O had a certain inhibitory effect on acyl-coenzyme A [22]. However, no other biological activities of pyripyropene O have been reported. In this study, we first found that pyripyropene O had a significant inhibitory effect on the cell viability of PC-3 prostate cancer cells through compound pre-screening. It was then further found that the inhibition of prostate cancer cell viability was due to the induction of apoptosis in prostate cancer cells. In addition, we showed that pyripyropene O induces apoptosis in prostate cancer cells by targeting the YY1/DR5 axis. This study identifies a potential prostate cancer drug and provides a new perspective on the treatment of prostate cancer.

## 2. Results

### 2.1. Pyripyropene O Suppressed Prostate Cancer Cell Proliferation

**Figure 1:**
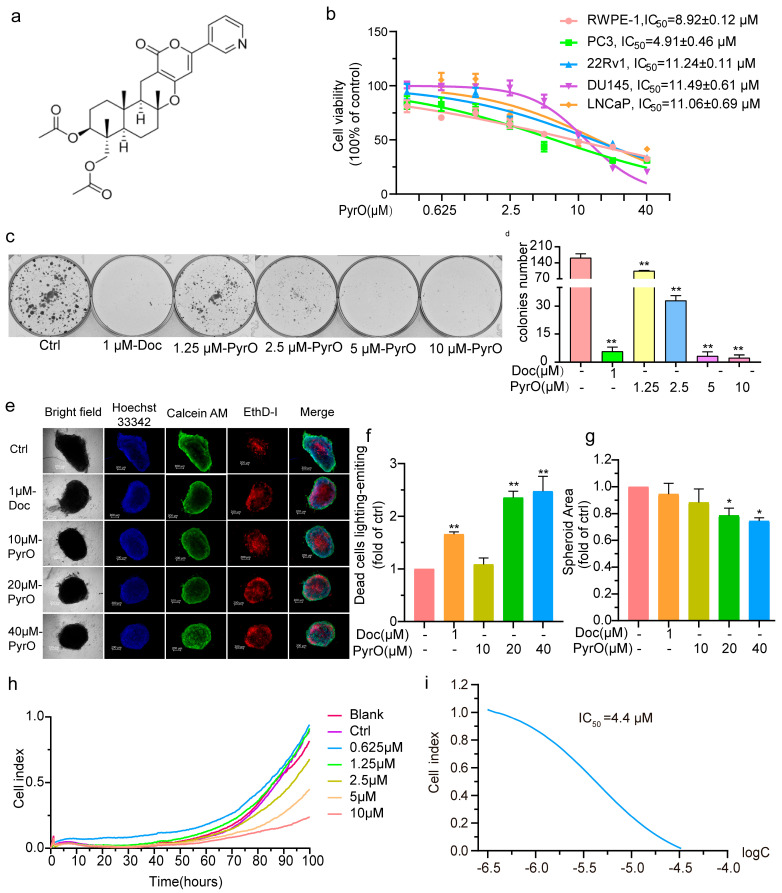
Effects of Pyripyropene O (PyrO) on the proliferation and migration of prostate cancer cells. (**a**) Chemical structural formula of Pyripyropene O (PyrO). (**b**) Effect of PyrO on cell viability. Cell viability was measured by the MTT assay after treatment of prostate cancer cells and normal prostate epithelial cells with different concentrations of PyrO (0–40 μM) for 72 h. (**c**,**d**) Effect of PyrO on clone formation in PC-3 cells. PC-3 cells were treated with PyrO (0–10 μM) for 14 days, then stained with crystal violet, and then photographed to observe the effect of PyrO on the ability of PC-3 cells to form clonal colonies. (**e**–**g**) Effect of PyrO on PC-3 three-dimensional proliferation. After treating PC-3 cell spheres inoculated in U-plates with PyrO (0–40 μM) for 72 h, live and dead cells were stained, and then the size of the cell spheres as well as the number of live and dead cells were observed by fluorescence microscopy. (**h**,**i**) Effect of PyrO on PC-3 cell migration. PC-3 cells were inoculated in CIM-plate 16 (5665817001, Agilent) plates, and the cells were treated with PyrO (0–10 μM) for 72 h. The migration of the cells was monitored by real-time cell analysis (RTCA). * *p* < 0.05, ** *p* < 0.01 vs. Ctrl.

We examined the effect of PyrO (Figure 1a) on the viability of prostate cancer cells using the MTT assay to preliminarily assess its anti-prostate cancer potential. The prostate cancer cells we used included PC3, 22Rv1, LNCaP, and DU145. The IC_50_s of PyrO inhibition of the above cells were 4.91 ± 0.46 μM, 11.24 ± 0.11 μM, 11.06 ± 0.69 μM, and 11.49 ± 0.61 μM (Figure 1b). We also examined the effect of PyrO on the viability of normal prostate RWPE-1 cells with an IC_50_ of 8.92 ± 0.12 μM (Figure 1b). From the above data, it can be seen that PyrO had the strongest inhibitory effect on the PC-3 cell viability of the prostate cancer cells and normal prostate cells tested. This suggests that PyrO is more sensitive to PC-3 cells. We therefore tested the effect of PyrO on prostate cancer proliferation using the plate clone formation assay and the three-dimensional cell sphere formation assay. The results showed that PyrO could significantly inhibit the proliferation of PC-3 cells on the plate and significantly reduce the number of PC-3 clonal colonies (Figure 1c,d). Similarly, PyrO also inhibited the formation of PC-3 cell spheres, and the size of PC-3 cell spheres in the PyrO-treated group was significantly smaller than that of the control group, and the number of dead cells in the spheres of the PyrO-treated group was significantly higher than that of the control group, as shown by staining (Figure 1e–g). In addition, we observed the effect of PyrO on PC-3 cell migration by tracking the migration of PC-3 cells after PyrO treatment in real-time using real-time cell analysis. The experimental results showed that the migration index of PC-3 cells was significantly reduced in a dose-dependent manner, and the IC_50_ of PyrO to inhibit PC-3 cell migration was 4.4 μM (Figure 1h,i). Taken together, these results suggest that PyrO has a significant inhibitory effect on prostate cancer cell viability and that this inhibitory effect, in turn, leads to the inhibition of both cell proliferation and migration.

### 2.2. Pyripyropene O Blocks the Cell Cycle and Induces Apoptosis in PC-3 Cells

**Figure 2:**
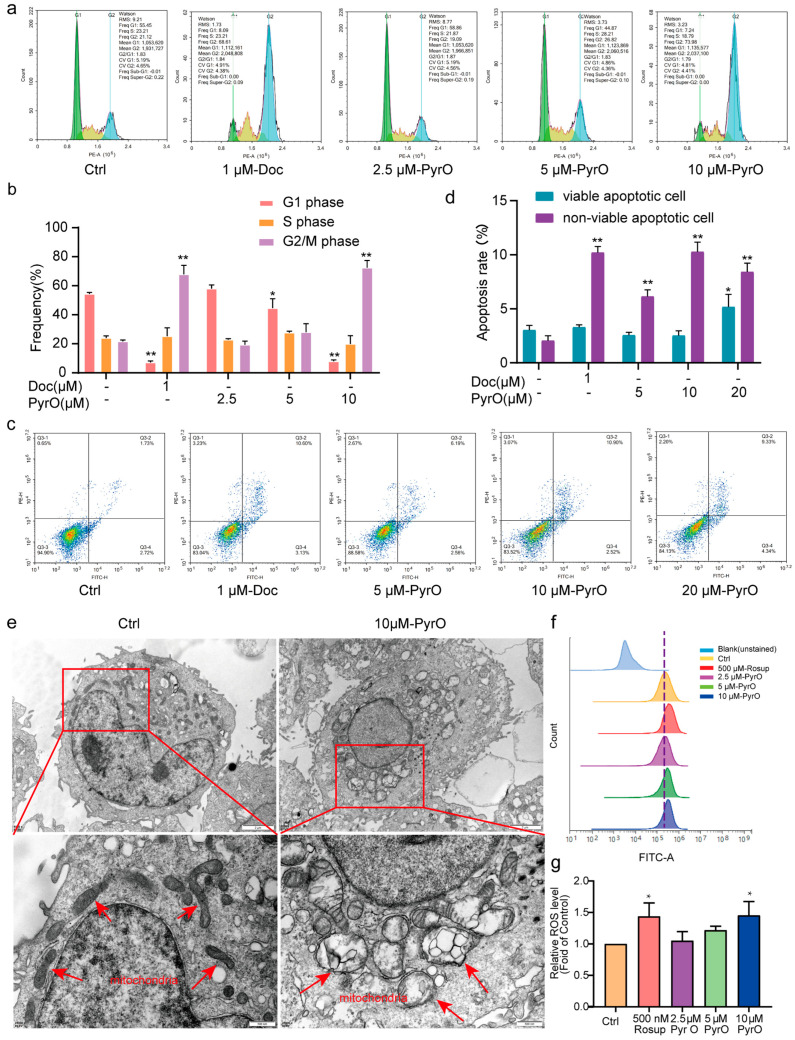
Effects of PyrO on PC-3 cell cycle and apoptosis. (**a**,**b**) PyrO blocked PC-3 cells in G2/M phase. Flow cytometry was used to detect changes in the distribution of the PC-3 cell cycle after treatment of PC-3 cells with different concentrations of PyrO for 48 h. (**c**,**d**) Effect of PyrO on apoptosis of PC-3 cells. After PyrO (0–20 uM) treatment for 48 h, the cells were stained with Annexin V-FITC/PI, and then the apoptosis was detected by flow cytometry. (**e**) Effect of PyrO on the internal structure of cells. 10 μM PyrO treated PC-3 cells for 48 h, then the cells were collected to observe the changes in the internal ultrastructure of the cells using transmission electron microscopy to analyze the mode of cell death. The red arrows in the image indicate the location of the mitochondria. (**f**,**g**) Effect of PyrO on intracellular ROS levels in PC-3 cells. DCFH-DA fluorescent probe combined with flow cytometry was used to detect the changes in intracellular reactive oxygen species (ROS) levels in PC-3 cells after treatment with different concentrations of PyrO (0–10 μM) for 48 h. * *p* < 0.05, ** *p* < 0.01 vs. Ctrl.

To further understand the cell fate of PC-3 cells after their viability was inhibited by PyrO, we analyzed the cell cycle and apoptosis of PyrO-treated PC-3 cells by flow cytometry. We found that the proportion of PyrO-treated PC-3 cells in the G2/M phase was much higher than that of the control group, like that of docetaxel (Figure 2a,b), indicating that PyrO blocked the PC-3 cell cycle at G2/M. We also found that PyrO induced apoptosis in PC-3 cells in a dose-dependent manner after 48 and 72 h of exposure to PyrO (Figure 2c,d and Figure S1). To further confirm the occurrence of apoptosis, we used transmission electron microscopy to observe the morphological changes in the internal structure of PC-3 cells after PyrO treatment. Electron microscopy revealed that the mitochondria of PyrO-treated PC-3 cells were swollen, and the endoplasmic reticulum was dilated, and these changes were consistent with the early stages of apoptosis (Figure 2e). In addition, we also detected the intracellular reactive oxygen species content by flow cytometry, and the results showed that the reactive oxygen species level in PyrO-treated PC-3 cells was significantly higher than that in the control group (Figure 2f,g). Based on the above experimental results, it can be confirmed that PyrO induced the occurrence of apoptosis in PC-3 cells.

### 2.3. Pyripyropene O Induces Apoptosis by Targeting the YY1/DR5 Axis

**Figure 3:**
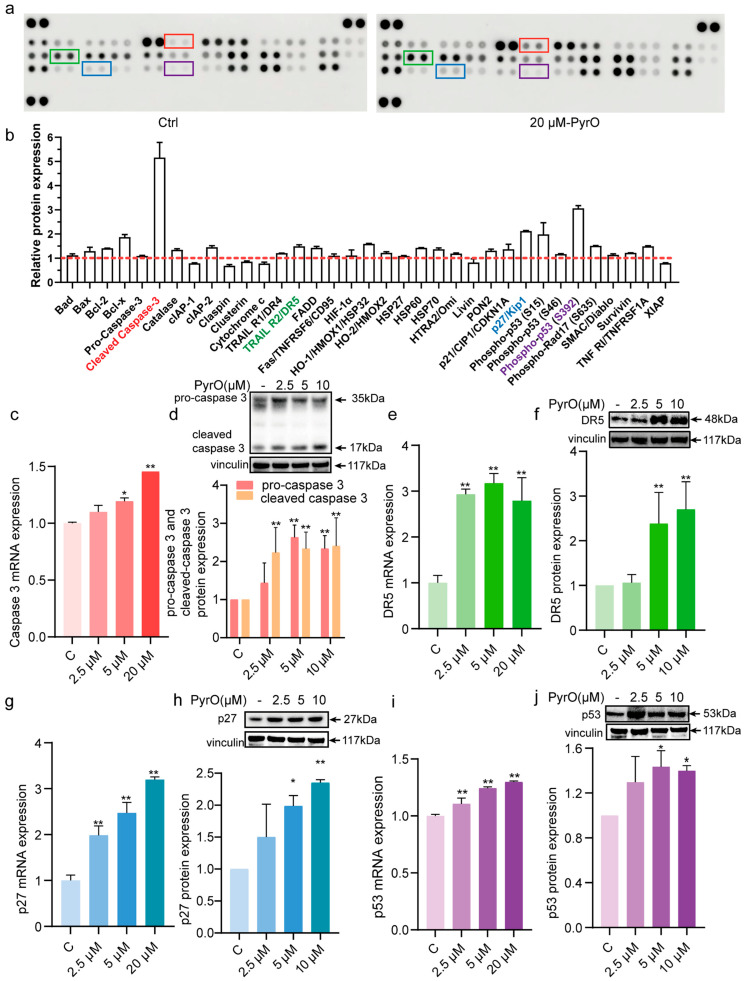
Pyripyropene O regulates the expression of apoptosis-related proteins and their mRNAs in PC-3 cells. (**a**,**b**) PC-3 cells were treated with PyrO (20 μM) for 48 h. Cells were collected and apoptosis-associated protein expression changes were detected using protein microarray kit. Red boxes indicate cleaved caspase 3 protein, green boxes indicate TRAIL R2/DR5 protein, blue boxes indicate p27/Kip1 protein, and purple boxes indicate phospho-p53(S392) protein. The red dotted line indicates Baseline (Negative Control). (**c**) After treating PC-3 cells with different concentrations of PyrO (0–20 μM) for 48 h, total RNA was collected, and the changes in caspase 3 mRNA expression were detected by qPCR after reverse transcription. (**d**) After treating PC-3 cells with different concentrations of PyrO (0–20 μM) for 48 h, total proteins were collected, and changes in caspase 3 protein expression were detected by western blot. (**e**) After treating PC-3 cells with different concentrations of PyrO (0–20 μM) for 48 h, total RNA was collected, and the changes in DR5 mRNA expression were detected by qPCR after reverse transcription. (**f**) After treating PC-3 cells with different concentrations of PyrO (0–20 μM) for 48 h, total proteins were collected, and changes in DR5 protein expression were detected by western blot. (**g**) After treating PC-3 cells with different concentrations of PyrO (0–20 μM) for 48 h, total RNA was collected, and the changes in p27 mRNA expression were detected by qPCR after reverse transcription. (**h**) After treating PC-3 cells with different concentrations of PyrO (0–20 μM) for 48 h, total proteins were collected, and changes in p27 protein expression were detected by western blot. (**i**) After treating PC-3 cells with different concentrations of PyrO (0–20 μM) for 48 h, total RNA was collected, and the changes in p53 mRNA expression were detected by qPCR after reverse transcription. (**j**) After treating PC-3 cells with different concentrations of PyrO (0–20 μM) for 48 h, total proteins were collected, and changes in p53 protein expression were detected by western blot. * *p* < 0.05, ** *p* < 0.01 vs. Ctrl.

To further understand how PyrO induces apoptosis in PC-3 cells, we detected the changes of apoptosis-related proteins after PyrO (20 μM) treatment of PC-3 cells using human protein microarray. The results showed that PyrO had a significant effect on the expression of cleaved-caspase 3, DR5, p27, and p53 among the apoptotic proteins. The levels of apoptosis-related proteins cIAP-2, FADD and stress-related proteins (heat shock proteins, catalase) were also synchronously increased. This result suggests that PyrO treatment may activate both pro-apoptotic signaling and anti-apoptotic/stress protective mechanisms. (Figure 3a,b). To verify this result, we further examined the expression of caspase 3, DR5, p27 and p53 at both gene and protein level using qPCR and Western blotting. The results showed that caspase 3 mRNA expression was significantly upregulated, while full-length caspase 3 protein levels decreased and cleaved caspase 3 increased (Figure 3c,d). These changes in caspase 3 confirm the initiation of apoptosis in the prostate cancer cell at the molecular level. In addition, DR5, p27 and p53 were consistently upregulated at both mRNA and protein levels (Figure 3e–j). These experimental findings suggest that PyrO-induced apoptosis is closely related to the above-mentioned proteins.

**Figure 4:**
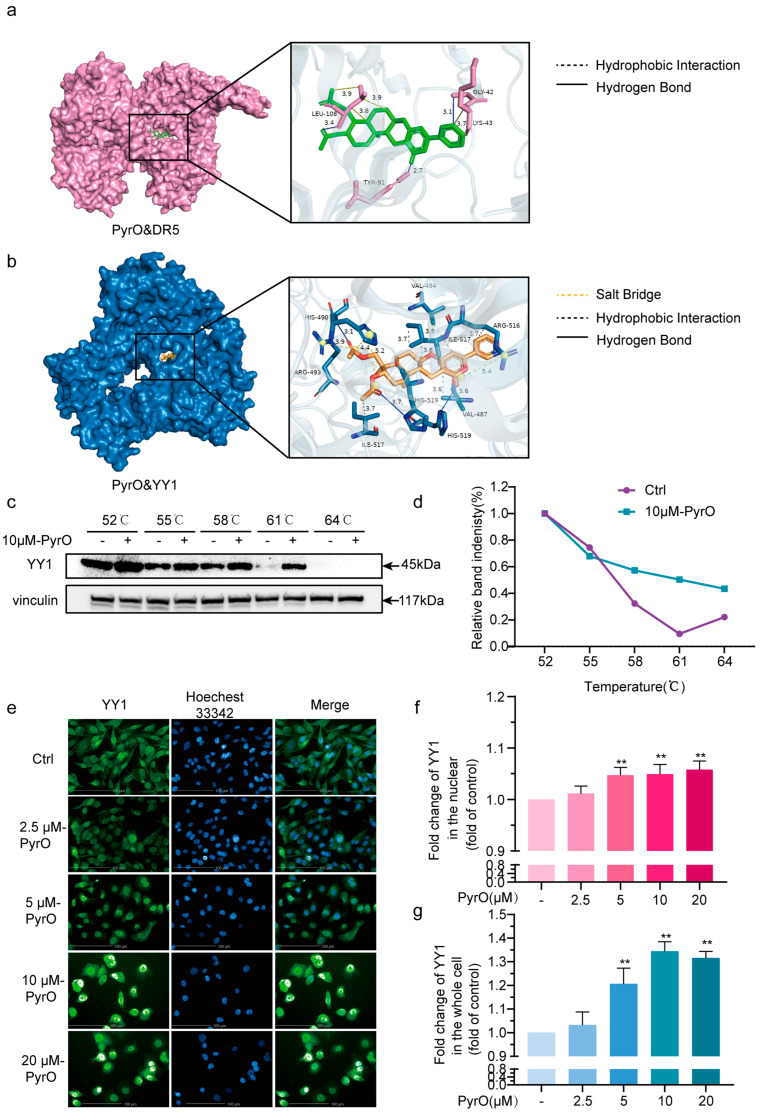
Pyripyropene O targets the YY1/DR5 axis to induce apoptosis. (**a**) Molecular docking technique was used to reveal the specific binding mode of PyrO to DR5 protein and its intermolecular interactions. (**b**) Molecular docking technique was used to reveal the specific binding mode of PyrO to YY1 protein and its intermolecular interactions. (**c**,**d**) Effect of PyrO on the heat stability of YY1 protein. 10 μM PyrO treated PC-3 for 8 h. Cells were collected, and total protein was collected before analysing the heat stability of the protein using cellular thermal shift assay (CETSA). (**e**–**g**) Effect of PyrO on nuclear translocation of YY1. PC-3 cells were treated with PyrO for 20 h. Immunofluorescence staining was used to detect the localization of YY1 protein in the cells. ** *p* < 0.01 vs. Ctrl.

Death receptor 5 (DR5) is a member of the tumor necrosis factor receptor (TNFR) superfamily and can trigger the signal transduction process of apoptosis when specific ligands (e.g., TRAIL) bind to DR5 on the cell surface [23]. The P53 gene is an important tumor suppressor gene that is activated when cells are subjected to DNA damage or other stressful stimuli [24]. The P53 protein initiates the apoptotic program by regulating the expression of apoptosis-related proteins [25]. P27 is one of the apoptotic proteins that receives regulation from P53 and promotes apoptotic cell death [26]. Given that PyrO alters the expression of apoptosis-related proteins as described above and the association between these proteins. We hypothesize that PyrO triggers prostate cancer cells to undergo exogenous apoptosis via DR5. We therefore used molecular docking to calculate the binding affinity of PyrO to the DR5 protein. Unfortunately, we found that the binding affinity of PyrO to DR5 protein was weak (Figure 4a, Table 1). However, qPCR, protein microarray and Western blot confirmed that DR5 mRNA and protein expression are indeed increased. Accordingly, we speculated that PyrO most likely binds to one or more transcription factors of DR5, and by regulating the transcription level of this or these transcription factors, it regulates the mRNA and protein expression of DR5. We then obtained 5 transcription factors of DR5 from the GeneCards website and calculated the binding affinity of PyrO to these transcription factors by molecular docking (Table 1). Molecular docking results showed that PyrO binds to the YY1 protein in three modes. One mode is that PyrO forms hydrogen bonds with HIS-490 and HIS-9 amino acid residues; the second mode is that PyrO forms hydrophobic interactions with HIS-490, ARG-493 and ARG-516 residues to form salt bridges; and the third mode is that PyrO interacts hydrophobically with VAL-494, ILE-517 and VAL-487 residues (Figure 4b). Based on these findings, we investigated the intracellular binding of PyrO to YY1 protein by cellular heat transfer assay. The results showed that PyrO stably increased the thermal stability of YY1 protein (Figure 4c,d). This experimental result indicates that PyrO binds to YY1 protein in prostate cancer cells. We then followed the distribution of YY1 protein in cells by cellular immunofluorescence staining and found that PyrO significantly promoted YY1 nuclear translocation (Figure 4e–g). Taking together with the regulatory effect of PyrO on DR5 shown in Figure 3, we can conclude that PyrO promotes YY1 nuclear translocation by binding to YY1 protein, regulates mRNA expression and protein expression of the YY1 target gene DR5, and thus regulates the onset of apoptosis.

### 2.4. Virtual Pharmacokinetic Characterization of Pyripyropene O

The predicted vivo absorption of PyrO is shown in Table 2. PyrO excelled in intestinal permeability with a Caco-2 value of −5.028 log cm/s (better than the threshold value of −5.15) and MDCK passive permeability of 2.1 × 10^−^⁵ cm/s (>20 × 10⁶ cm/s), and was hardly a Pgp substrate (probability 0.001), but has a high probability of 0.996 as a Pgp inhibitor; human intestinal absorption (HIA) >30% but oral bioavailability is low (F_20%_ probability 0.51, F_30%_ probability 0.988), suggesting that it may not be suitable for oral administration.

The predicted in vivo distribution of PyrO is shown in Table 3. The plasma protein binding (PPB) of PyrO was 80.78% (<90%), the volume of distribution (VD) was 1.0 L/kg (normal range 0.04–20 L/kg), which possessed some blood-brain barrier penetration ability (probability 0.176), and the plasma free fraction (Fu) was 21.33% (>20%), suggesting that it had a good ability of transmembrane diffusion and was widely distributed in vivo.

The predicted vivo metabolism of PyrO is shown in Table 4. PyrO inhibits CYP3A4 with a probability of 0.81 (high risk), which may trigger drug-drug interactions, but all other CYP enzymes (1A2/2C19/2C9/2D6) with a probability of inhibition <0.5; low substrate probability and high metabolic stability for all CYP enzymes.

The predicted vivo excretion of PyrO is shown in Table 5. The clearance (CL) of PyrO was 2.193 mL/min/kg (<5, low clearance), suggesting that it is susceptible to accumulation in the body; the probability of a half-life (T_1/2_) of >3 h was only 0.278, which predicts a short half-life.

We performed the toxicity prediction of PyrO by using ADMETlab 2.0, and the results are shown in Table 6. PyrO was not genotoxic (AMES: 0.011), carcinogenic (0.067), eye irritant/corrosive (0.003/0.009), or acutely toxic in rats (0.104), but there was significant hepatotoxicity (H-HT 0.777, DILI 0.822), respiratory toxicity (0.929), and some cardiotoxicity (hERG 0.583), skin sensitization (0.566) and risk of FDAMDD toxicity (0.454).

Overall, PyrO exhibited good drug permeability and broad tissue distribution properties, but its oral bioavailability was low. The compound may also exhibit some CYP3A4 enzyme inhibitory activity, which may trigger clinically relevant drug interactions. Notably, toxicological predictions showed a dose-dependent risk of hepatic parenchymal injury and respiratory toxicity. Therefore, to further develop this marine natural product, it is necessary to establish a precise drug delivery scheme based on population pharmacokinetics and strictly control the blood concentration; to develop new drug delivery systems such as slow-release microspheres or nanoliposomes to enhance the bioavailability; and to improve the efficacy/toxicity ratio through structural modification.

### 2.5. In Vivo Anti-Prostate Cancer Activity of Pyripyropene O

**Figure 5:**
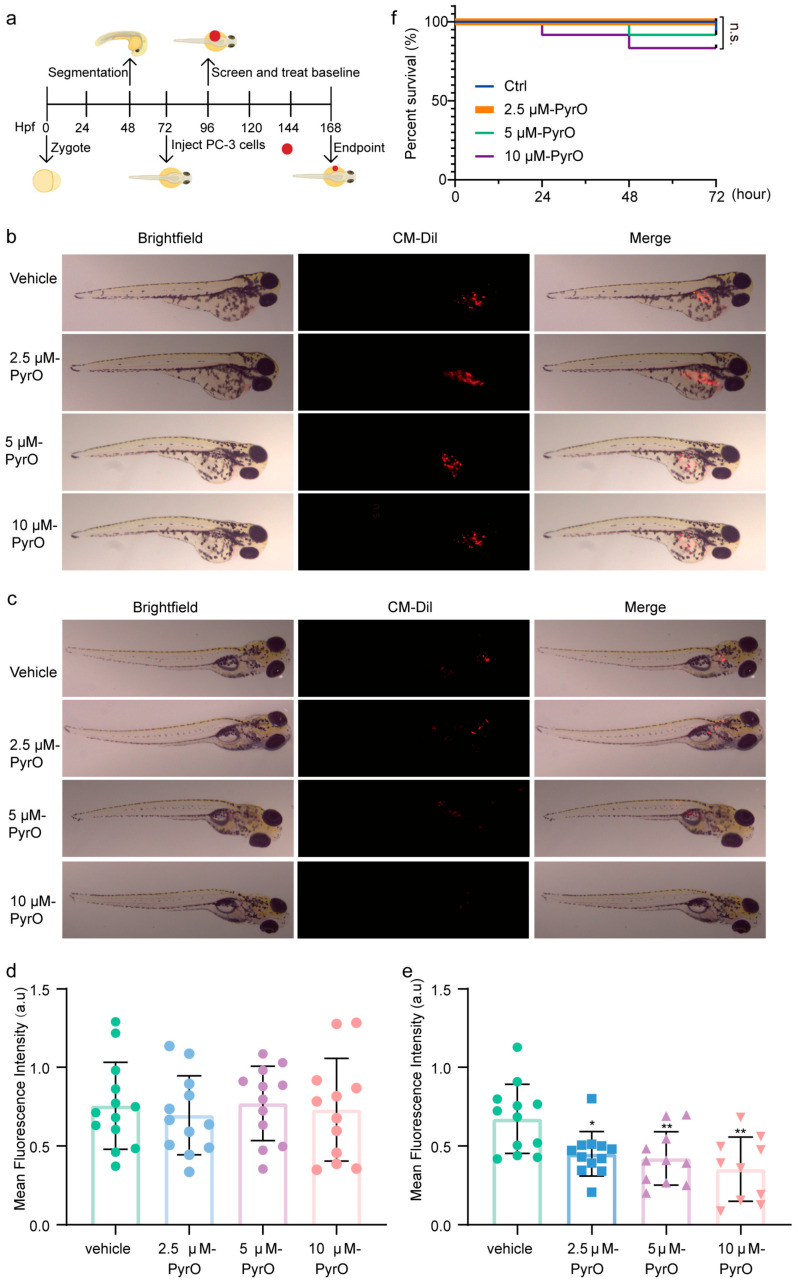
Pyripyropene O inhibits the growth of xenograft prostate cancer PC-3 cells in zebrafish. (**a**) Procedure for establishing a zebrafish xenograft tumor model. (**b**) Distribution of prostate cancer PC-3 cells before administration of Pyripyropene O in zebrafish xenografts. (**c**) Distribution of prostate cancer PC-3 cells in zebrafish after 72 h treatment with different concentrations of Pyripyropene O. (**d**) Intensity of fluorescence in zebrafish in each group before drug administration. (**e**) Fluorescence intensity in zebrafish from each group after 72 h of PyrO treatment. (**f**) Zebrafish survival rate after Pyripyropene O administration. * *p* < 0.05, ** *p* < 0.01 vs. vehicle.

Since PyrO induces apoptosis and inhibits survival, proliferation and migration of prostate cancer cells at the cellular level. We then predicted the in vivo absorption, distribution, metabolism, excretion process and potential toxic effects of PyrO by using ADMETlab 2.0 software. The data from these experiments proved that PyrO is a molecule worthy of further study. Zebrafish are widely used in various fields due to their unique biology and high genetic similarity to humans [27]. Zebrafish have been used to establish various tumor models to evaluate the therapeutic efficacy of agents [19,28]. To evaluate the anti-prostate cancer effect of PyrO in animals with minimal compounds, we used a zebrafish xenograft tumor model (Figure 5a). After pre-stained prostate cancer cells were injected into zebrafish, zebrafish with concentrated fluorescence areas and similar fluorescence intensities were screened and divided into four groups (Ctrl, 2.5 μM-PyrO, 5 μM-PyrO, 10 μM-PyrO), at which time the average fluorescence intensities of each group of zebrafish were 0.76 ± 0.28, 0.70 ± 0.25, 0.77 ± 0.24, 0.73 ± 0.33 a.u, respectively (Figure 5b,d). The zebrafish in each group were then exposed to different concentrations of PyrO for 72 h. The results showed that the mean fluorescence intensity of zebrafish decreased significantly with the increase of PyrO concentration, and the mean fluorescence intensities of zebrafish in each group were 0.65 ± 0.23, 0.45 ± 0.14, 0.42 ± 0.17, and 0.35 ± 0.20 a.u, respectively. Moreover, compared with the control group, the mean fluorescence intensities of the administered groups decreased by 30.43%, 34.93%, and 45.50% (Figure 5c,e), indicating that PyrO inhibited the growth of zebrafish prostate cancer cells. We monitored the survival of zebrafish during the administration period. Except for the 2.5 μM PyrO group, 1–2 zebrafish died in all groups (Figure 5f). However, there was no significant difference in the overall survival of zebrafish in each group during the administration period, suggesting that PyrO is safe and effective in the concentration range we tested.

## 3. Discussion

Pyripyropene O is a natural small molecule of marine origin, and studies on its pharmacological activity are extremely limited, with only a few relevant reports in 1996 and 2011, and the research field is relatively restricted [22,29]. Our team has long been engaged in the discovery of anti-prostate cancer natural products and their mechanism of action. In the present study, we found that pyripyropene O significantly inhibited the viability of prostate cancer cells. Further studies also showed that pyripyropene O induced apoptosis of prostate cancer cells and that pyripyropene O promoted apoptosis of prostate cancer cells by activating the expression and function of DR5 through binding to the transcription factor YY1.

Yin Yang 1 (YY1) is a common zinc finger protein transcription factor that is widely expressed in human tissues and is involved in the control of a variety of cellular mechanisms, with dual regulatory roles of transcriptional activation and transcriptional repression [30]. Death Receptor 5 (DR5) is a member of the tumor necrosis factor receptor superfamily that initiates apoptotic signaling pathways upon binding to specific ligands [31,32]. YY1 is essential for the regulation of DR5 expression [33,34]. It has been shown to negatively regulate DR5 transcription and upregulate DR5 expression in tumors by binding to specific sequences in the promoter region of the DR5 gene [35,36]. Once DR5 expression is upregulated, binding to the appropriate apoptotic ligand (e.g., TRAIL) can more effectively activate the downstream apoptotic signaling cascade, inducing tumor cells to undergo apoptosis and thereby inhibiting tumor growth and proliferation [33,37,38,39]. Therefore, targeting the YY1/DR5 axis may be an effective anti-cancer strategy. Studies reported that YY1 is associated with prostate cancer-specific metabolic profiles, with overexpression of YY1 in prostate tissue and increased mitochondrial energy metabolism activity when normal prostate tissue progresses to cancer [40,41]. It has also been found that Yin Yang 1 promotes neuroendocrine differentiation of prostate cancer cells through a non-classical WNT pathway [42]. In addition, it has been proposed that the YY1 complex in M2 macrophages promotes prostate cancer progression by upregulating IL-6 [43]. These findings provide new perspectives for the specific diagnosis and treatment of prostate cancer.

Systematic studies have demonstrated that a variety of chemical entities exhibit significant therapeutic potential by targeting YY1 proteins, and their molecular mechanisms are characterized by a high degree of diversity. In cancer therapy, oxymatrine inhibits iron death in hepatocellular carcinoma through the SIRT1/YY1/GPX4 axis [44], diosbulbin B targets YY1 to induce cell cycle arrest and apoptosis in non-small cell lung cancer [45], JAC1 inhibits the proliferation of triple-negative breast cancer by inhibiting YY1/HSF1/p-Akt signaling [46], the gold(III) porphyrin complex Gold-2a inhibits breast cancer WNT1 by enhancing YY1 promoter binding [47], and the nitric oxide donors DETANONOate and GIT-27NO reverse tumor resistance by interfering with NF-κB/Snail/YY1/RKIP or directly inhibiting YY1 [48,49]. In addition, picroside II inhibits TGFβ1 transcriptional activity via activation of YY1 to alleviate renal fibrosis [50], liquiritin treats psoriasis by modulating the YY1/RBP3 axis [51], quercetin ameliorates diabetic hepatic lipid accumulation via the mTOR/YY1 pathway [52], berberine protects against endothelial via AMPK/NF-κB/YY1 injury [53], and betaine prevented obesity via miR-378a/YY1 [54]. Analysis of the structures of the above compounds showed that although there are significant structural differences between the above compounds and Pyripyropene O, they all exhibit typical bioactive molecular features: most of them contain nitrogen heterocyclic structural domains or complex multicyclic skeletons, and the natural product source accounts for a prominent proportion. Specifically, their ring configurations are heterogeneous in terms of aromatic rings, glycosidic ligands and metal complexes, which provides a rich chemical space for the development of YY1-targeted drugs.

Previous studies have shown that YY1 negatively regulates DR5 expression, as evidenced by the fact that inhibition of YY1 expression promotes DR5 expression [37,55]. However, the present study found that the regulation of DR5 by YY1 was different from previous studies. In this study, we found that pyripyropene O significantly inhibited the viability of prostate cancer PC-3 cells. Specifically, at the cellular level, pyripyropene O induced apoptosis in PC-3 cells, thereby inhibiting cell proliferation and migration. At the molecular level, we found that pyripyropene O upregulated DR5 protein expression using Human Apoptosis Protein array, Western Blot and qPCR, suggesting that it may induce apoptosis by regulating DR5. Unfortunately, in molecular docking experiments, we found that pyripyropene O is very unlikely to bind directly to DR5, whereas it is very likely to bind to YY1, a transcription factor of DR5. Therefore, we hypothesised that pyripyropene O may play a role in inducing apoptosis by targeting YY1 to regulate DR5 expression. We then confirmed our speculation by molecular docking, cellular thermal shift assay and cellular immunofluorescence assay. In addition, zebrafish in vivo experiments verified the anti-prostate cancer effects of pyripyropene O in animals. In conclusion, the marine natural product pyripyropene O induces apoptosis of prostate cancer PC-3 cells by targeting the YY1/DR5 axis, resulting in an anti-prostate cancer effect.

This study reveals that Pyripyropene O promotes YY1 nuclear translocation by directly binding to the transcription factor YY1, thereby upregulating the expression of the YY1 target gene DR5, which then initiates apoptosis. However, this study has some limitations. Firstly, we tested the effect of pyripyropene O on cell viability in four prostate cancer cell lines, and the experiments showed that pyripyropene O had the most significant inhibitory effect on PC3 cells. However, among the four cells we tested, both DU 145 and LNCaP had higher YY1 gene expression than PC-3 (Figure S2). The reason why pyripyropene O is more sensitive to cells with low YY1 expression needs to be further investigated. On the other hand, the total amount of pyripyropene O obtained from our current extraction experiment was limited. At the animal level, we chose the zebrafish model to evaluate the in vivo anti-prostate cancer effect of pyripyropene O as the amount of compound required for the zebrafish model is relatively small. If sufficient compound is available, a patient-derived xenograft tumour mouse model could then be performed to further validate the in vivo effect of pyripyropene O. In addition, the analysis results of the ADMETlab 2.0 software indicated that pyripyropene O may be hepatotoxic and its oral bioavailability is low, which poses a challenge for further research and clinical application of pyripyropene O. The challenges faced by pyripyropene O may be further overcome by modification of the chemical structure or formulation technology. In addition, more research is needed to determine the dosage and delivery method of Pyripyropene O, as well as to evaluate its safety.

In conclusion, Pyripyropene O is not yet a perfect anti-prostate cancer molecule. However, pyripyropene O is a natural marine product that can bind directly to the transcription factor YY1 and induce apoptosis in prostate cancer cells by targeting the YY1/DR5 axis. This finding suggests that marine natural products contain molecules with diverse biological activities and novel mechanisms of action. Pyripyropene O may serve as a lead compound against prostate cancer and deserves further investigation. In addition, this study highlights the potential of marine natural products in anti-prostate cancer drug discovery, providing more opportunities for the development of new anti-prostate cancer drugs.

## 4. Materials and Methods

### 4.1. Compound Preparation

The soft coral BH2, obtained from Beihai, Guangxi Province, China during November 2018, yielded the fungal strain *Aspergillus fumigatus* SCSIO41220. Through morphological examination and sequence analysis of the internal spacer (ITS) regions of the rDNA, it was determined that this strain belongs to *Aspergillus fumigatus*. A voucher specimen has been deposited in the CAS Key Laboratory of Tropical Marine Bio-resources and Ecology, South China Sea Institute of Oceanology, under the Chinese Academy of Sciences in Guangzhou, China. The marine fungus *Aspergillus fumigatus* SCSIO41220 was subjected to large-scale fermentation using a rice medium. A small amount of slant culture was used for seed fermentation (MA medium: maltose 15 g, sea salt 24 g, distilled water 1 L), with 200 mL medium in a 500 mL Erlenmeyer flask, and cultured at 25 °C on a shaking incubator (180 rpm). After 5 days of cultivation, 10 mL of the seed fermentation broth was transferred to sterilized conical flasks containing rice medium (rice 200 g, water 220 mL, resulting in a concentration of 32 g/L), with a total of 78 flasks, and fermented under static conditions at a temperature of 25 °C for 50 days. The fermentation broth was extracted four times with ethyl acetate, and the solvent was recovered to yield the crude extract (163.0 g).

The crude extract (163.0 g) was subjected to medium-pressure normal-phase silica gel column chromatography for separation. Initially, a gradient elution was performed using a mixture of petroleum ether and ethyl acetate with a ratio of 100%/0% to 50%/50%. Subsequently, a gradient elution was carried out using a mixture of dichloromethane (PE) and ethyl acetate (EA) with a ratio of 90%/10%~0%/100%, followed by methanol. Fractions were then identified and combined based on thin-layer chromatography (TLC) and detected using a diode array detector (DAD), resulting in eight fractions, designated as Fr.1~Fr.8. Subsequently, each of the fractions Fr.5, Fr.6, Fr.7, and Fr.8 were subjected to gradient elution using an ODS column with a methanol/water system (5~100%). This process yielded the following sub-fractions: Fr.5–1~Fr.5–37, Fr.6–1~Fr.6–33, Fr.7–1~Fr.7–9, and Fr.8–21. Among these fractions, Fr.6–10 was also purified using semi-preparative HPLC with the following conditions: a methanol/water mixture with a ratio of 64/36, at a flow rate of 2.5 mL/min, resulting in the isolation of pyripyropene O (9.32 mg, t*_R_* = 22 min, Purity: >95%).

### 4.2. Reagents and Antibodies

RPMI 1640 medium, DMEM F12 medium, DMEM medium, and Keratinocyte-SFM medium were purchased from Gibco (Cat# C11875500BT, Cat# C11330500BT, Cat# C11995500BT, Cat# 10724011). Fetal bovine serum (Cat# F0193) was obtained from Sigma (Louis, MO, USA). Thiazolyl Blue Tetrazolium Bromide (Cat# M8180), Crystalline Violet Staining Solution (Cat# G1063), and DMSO (Cat# D8371) were from Solarbio Life Sciences (Beijing, China). nuPAGETM 10% Bis-Tris gels (Cat# NP0302BOX, Cat# NP0303BOX), eBioscience™ Annexin V-FITC Apoptosis Detection Kit (Cat# BMS500FI-300), FxCycle™ PI/RNase Stain (Cat# F10797), and CellTracker™ CM-DiI (Cat# C7000) were from Invitrogen (Carlsbad, CA, USA). Docetaxel (Cat# S1148) was purchased from Selleck.cn (Shanghai, China). ROS Reactive Oxygen Species Assay Kit (Cat# R6033-A) was purchased from UElandy (Suzhou, China). Live/Dead Cell Staining Kit (Cat#PF00008) and antibodies (DR5, Cat#15497-1-AP; Caspase3, Cat#19677-1-AP; p27, Cat#67355-1-Ig; p53, Cat#60283-2-Ig; YY1, Cat#66281-1-IG; vinculin, Cat#26520-1-AP) were purchased from Proteintech Group (Wuhan, China).

### 4.3. Cell Culture

Human prostate cancer cell lines (LNCaP, 22Rv1, PC-3, DU145) and normal prostate cells RWPE-1 were purchased from the Chinese Academy of Sciences (CAS) Cell Bank/Stem Cell Bank (Shanghai, China), and the culture system of each cell line strictly followed the standardized operation procedures of the institution. All cells were routinely cultured in a 5% CO_2_, 37 °C incubator. Among them, RWPE-1 cells were cultured using keratinocyte-SFM medium containing EGF human recombinant (10450013, Gibco), supplemented with 20–30 μg/mL bovine pituitary extract (13028014, Gibco) and 0.1% gentamicin/amphotericin B solution (500640, Gibco). Tumor cell lines were cultured under the following conditions: LNCaP and 22Rv1 were cultured in RPMI 1640 medium, PC-3 was cultured in DMEM/F12 medium, and DU145 was cultured in DMEM medium, each supplemented with 10% (*v*/*v*) South American-made fetal bovine serum (Sigma-Aldrich, Louis, MO, USA) and 1% penicillin/streptomycin solution. Before the experiment, we confirmed that all cell lines met the following quality control criteria: mycoplasma-negative results and cell identity verified by STR profiling.

### 4.4. Cell Viability and Growth Analysis

The MTT experiment was performed in the same way as in our previous study [56]. In brief, PC-3 cells (5 × 10^3^−1 × 10^4^ cells/well) were treated with PyrO for 72 h. MTT reagents were added and incubated for 4 h. IC_50_ was calculated using GraphPad Prism 8.0. values. For colony formation assays, PC-3 cells (1 × 10^3^ cells/well) were treated with PyrO (1.25, 2.5, 5, and 10 μM) for one week, fixed with 4% tissue fixative, stained with crystal violet, and analyzed for the number of colonies using the GelCount system (GelCount, Oxford Optronix). For three-dimensional sphere formation analysis, 200 μL of a mixed suspension containing PC-3 cells (1 × 10^4^ cells/well) and 2% matrix gel was inoculated into a 96-well U-bottom plate (174925, Thermo). After centrifugation to allow cell aggregation, the cells were incubated for 24 h to form spheres. The cells were treated with PyrO (10, 20, 40 μM) and docetaxel (1 μM) for 72 h. Cells were stained for 4 h and washed three times with PBS containing 5% fetal bovine serum according to the kit instructions (Viability/Cytotoxicity Assay Kit for Animal Live & Dead Cells, Proteintech, Cat# PF00008). Spherical morphology and distribution of live and dead cells were observed using an inverted fluorescence microscope (Zeiss Axio Vert. A1, Oberkochen, Germany). The positive control used in the study was docetaxel. Docetaxel is a microtubule stabilizer that targets microtubule proteins and is a first-line chemotherapy drug for the treatment of prostate cancer [57]. All experiments were repeated three times independently.

### 4.5. Cell Migration Assay

Different concentrations (0.625, 1.25, 2.5, 5, 10 μM) of PyrO working solution were prepared using DMEM/F12 medium containing 10% fetal bovine serum. Add 165 μL of PyrO working solution to the lower chamber of the CIM-plate 16 (5665817001, Agilent, Santa Clara, CA, USA) plate and set up two replicate wells for each concentration. The plate was then assembled, and 30 μL of serum-free medium was added to the upper chamber wells and equilibrated at 37 °C for 1 h. Baseline impedance values were collected using a real-time cell analyzer (xCELLigence RTCA DP instrument, Agilent, Santa Clara, CA, USA). 100 μL of serum-free PC-3 cells (2.5 × 10^4^ cells/well) were inoculated into the upper chamber wells, and then 14.4 μL of PyrO solution (6.25, 12.5, 25, 50, and 100 μM) was added to make the concentration of PyrO in the upper and lower chambers consistent, and then the cells were allowed to rest at room temperature for 30 min, and then transferred to the real-time cell analyzer for continuous recording.

### 4.6. Apoptosis and Cell Cycle Assay

PC-3 cells (2 × 10^5^ cells/well) were inoculated into 6-well plates and cultured for 48 h. Cells were treated with 0.1% (*v*/*v*) DMSO, 1 μM docetaxel and the indicated concentrations of PyrO for 48 h and then harvested. Apoptosis was detected using the eBioscience™ Annexin V—FITC Apoptosis Detection Kit (BMS500F1—300, Invitrogen, Carlsbad, CA, USA); cell cycle distribution was detected by staining with FxCycle™ PI/RNase staining solution (F10797, Invitrogen). The results were finally analyzed by flow cytometry (NovoCyte, Agilent, Santa Clara, CA, USA), and the experiments were independently repeated three times.

### 4.7. Transmission Electron Microscopy

PC-3 cells (5 × 10^6^ cells/dish) were inoculated into 100 mm dishes for 48 h and then treated with 10 μM PyrO for 48 h. Cells in the dishes were collected, pre-fixed with 2.5% glutaraldehyde and post-fixed with 1% osmium tetroxide, and then dehydrated through a gradient of acetone (30%→100%, and 100% concentration was changed three times) in sequence, and then embedded in Epon-812 embedding medium at the ratios of 3:1, 1:1, and 1:3, and then prepared into 60–90 nm ultrathin sections by gradient infiltration of pure embedding medium and transferred to copper mesh. After gradient permeation with Epon-812 embedding medium at 3:1, 1:1, 1:3 ratio, 60–90 nm ultrathin sections were prepared and transferred to copper mesh after embedding with pure embedding medium, and finally the samples were double stained with uranyl acetate (10–15 min) and lead citrate (1–2 min) to complete the sample preparation. The sections were then examined and photographed using a JEM-1400-FLASH transmission electron microscope.

### 4.8. Cellular Reactive Oxygen Species Assay

PC-3 cells (3 × 10^5^ cells/well) were inoculated in 6-well plates. After the morphology of the cells returned to normal in the culture wells, cells were treated with a series of PyrO (2.5, 5, 10 μM) solutions for 24 h. The cells were then pre-treated with a positive control according to the ROS Reactive Oxygen Specific Assay Kit (R6033-A, UElandy, Suzhou, China), and the cells were incubated with the positive control Rosup (500 μM) for 1 hr at 37 °C. Cells from all treatment groups were then collected and stained with DCFH-DA (1:2000) for 30 min in the dark, followed by two washes with PBS buffer. The relative intensity of ROS in live cells was determined by flow cytometry (FongCyte™, Challen Bio, Beijing, China).

### 4.9. Proteome Profiler Human Apoptosis Array

PC-3 cells (5 × 10^6^ cells/well) were inoculated into 100 mm dishes and cultured for 48 h, then the cells were treated with 0.1% DMSO and 20μM PyrO for 48 h, and then the cells were harvested, proteins were extracted, and immunoblotting reactions were performed according to the Human Apoptosis Array Kit (ARY009, bio-techne) user manual. Finally, Chemidoc CD Touch (Bio-Rad, Hercules, CA, USA) was used to capture and analyze the immunoblotting images, and then Image Lab 6.0 (Bio-Rad, Chinese version) was used to further analyze the images to obtain greyscale data and assess the changes in the expression of various proteins.

### 4.10. Molecular Docking Study

The 2D structure SDF file of pyripyropene O was obtained from PubChem Compound Database https://www.ncbi.nlm.nih.gov/pccompound/ (accessed on 12 November 2024). The molecular structure file was then imported into Chem3D molecular modeling software (version 20.0), and energy minimization calculations were performed using the MM2 algorithm. Finally, the optimized 3D molecular structure was exported and saved in PDB format for subsequent molecular docking studies. Potential transcriptional regulators of DR5 proteins were screened by GeneCard database. Based on the PDB protein database, 3D models of a series of target proteins were constructed: the 3D structural models of DR5 (PDB ID: 2H9G), YY1 (PDB ID: 4C5I), AP1 (PDB ID: 1A02), NF-κB (PDB ID: 1SVC) and CEBP (PDB ID: 6DC0) were obtained from the X-ray diffraction data. The three-dimensional structure models of SP1 (PDB ID: 1VA1) and TP53 (PDB ID: 5HOU) were based on nuclear magnetic resonance (NMR) experimental data. In molecular docking analysis, AMdock was utilized to predict the binding mode and binding free energy of the natural compound PyrO with key target proteins such as DR5, YY1, SP1, AP1, TP53, NF-κB and CEBP. Based on the AMdock platform, the spatial conformation was optimized by calculating the ligand-receptor interaction force to identify the optimal binding mode and potential binding sites. The docking results were visualized and analyzed in three dimensions using PyMOL to further validate the binding sites, and molecular force types such as hydrogen bonding and hydrophobic interactions were resolved using the PLIP online platform.

### 4.11. Immunofluorescence Assay

The 96-well black plates were pretreated with 10% polylysine. Then, PC-3 cells (1 × 10^4^ cells/well) were inoculated into 96-well black plates and cultured for 24 h. The PC-3 cells were treated with different concentrations of PyrO (0, 2.5, 5, 10, 20 μM) for 20 h. The cells were then fixed with 4% paraformaldehyde for 20 min, the cell membrane was permeabilized with 0.5% TritonX-100 for 15 min, and the cells were blocked with 1% BSA for 1 h. YY1 antibody was then added to the cells, and the cells and antibody were incubated at 4 °C overnight. The next day, the cells were treated with a fluorescent secondary antibody for 1 h. The nuclei were then stained with Hochest33342 for 5 min. Excess dye was washed away with PBS buffer, and images were captured at 20x magnification using the PerkinElmer High Content Screening System.

### 4.12. Realtime Fluorescence Quantitative PCR

PC-3 cells (2 × 10^6^ cells/well) were inoculated into six-well plates and cultured for 24 h. The cells were then treated with different concentrations of PyrO (0, 2.5, 5, 20 μM) for 48 h. Cells were collected, and RNA was extracted according to the instructions of TRIzol regent (15596026CN, Invitrogen). RNA was then reverse transcribed into cDNA according to the instructions of RevertAid™ Master Mix (M1631, Thermo Fisher Scientific, Waltham, MA, USA). Finally, a quantitative qPCR reaction was performed according to the instructions of PowerUp™ SYBR™ Green Master Mix (A25742, Invitrogen). qPCR reaction was carried out on a Roche LightCycler 96 instrument, and the data were analyzed using LightCycler 96 software. This experiment was repeated three times independently.

### 4.13. Western Blot

PC3 cells (5 × 10⁵cells/well) were inoculated into six-well plates and then treated with 2.5, 5 and 10 μM PyrO for 48 h. Cells were harvested and lysed with RIPA lysis solution (containing PMSF), and the supernatant was centrifuged to obtain total protein. Total protein concentration was determined using the BCA protein quantification kit (23225, Invitrogen), then protein samples were prepared with NuPAGE LDS buffer (NP0007, Invitrogen) and reducing agent (NP0009, Invitrogen) and denatured in a 70 °C metal bath. Proteins were separated by 10% Bis-Tris gel electrophoresis and then transferred to a PVDF membrane. The PVDF membrane was blocked with 5% skim milk for 1 h and then reacted with primary antibody at 4 °C overnight. The PVDF membrane was reacted with HRP-conjugated secondary antibody at room temperature for 1 h. Finally, the PVDF membrane was treated with Immobilon Western Chemiluminescent HRP Substrate solution (WBKLS0100, Millipore, EMD Millipore, Burlington, MA, USA), and the protein bands were detected using the Chemidoc CD Touch Imaging System (Bio-Rad, Hercules, CA, USA). The grey level and size of the protein bands were analysed using Image Lab 6.0 software (Bio-Rad, Chinese version).

### 4.14. Cellular Thermal Shift Assay (CETSA)

PC-3 cells (5 × 10^6^ cells/dish) were seeded in 100mm culture dishes. After cell attachment, PC-3 cells were treated with DMSO or PyrO (10 μM) for 8 h. The cells were harvested, and the PC3 cells were resuspended in PBS containing PMSF (PBS:PMSF = 100:1). Each cell sample was divided into five equal portions, and the cells were then lysed by repeated freeze-thawing three times between −80 °C and room temperature to obtain whole cell extracts. The cell extracts were then heated at the indicated temperatures (52 °C, 55 °C, 58 °C, 61 °C, 64 °C) for 3 min before centrifugation of the cell extracts, and the supernatant was the total cell protein. Total protein content was determined using the BCA Protein Quantification Kit (23227, ThermoFisher Scientific), and samples were prepared and subjected to Western blot as described above for the Western blot method.

### 4.15. ADMET Analysis of Pyripyropene O

ADMETlab 2.0 is a comprehensive online platform for accurate and comprehensive prediction of ADMET characteristics. We comprehensively evaluated the ADMET properties of compound PyrO through 23 ADME properties, 27 toxicity endpoints, and 8 toxicity group rules (751 substructures) of the platform.

### 4.16. Prostate Cancer Xenograft Model in Zebrafish

This study was approved by the Animal Welfare and Ethics Committee of Guangxi University of Chinese Medicine (DW20220525-090-08), and wild-type zebrafish of the AB line were used for the experiments. PC-3 cells were stained by 1 μg/mL CellTracker CM-DiI (C7000, Invitrogen), incubated at 37 °C with 5% CO_2_ for 5 min, then transferred to 4 °C and continued to be stained for 15 min, washed three times by D-PBS, and resuspended in DMEM F12 and stored on ice. Zebrafish were anesthetized with 0.1% ms-222, then placed on a 1.5% agarose gel, and approximately 200 PC-3 cells were injected into the yolk sac by microinjection. 24 h later, successfully injected zebrafish were screened under a fluorescence microscope and grouped into clusters (n = 12 or 13), then the zebrafish will be cultured in embryonic water containing different concentrations of PyrO for 72 h. Finally, fluorescence microimaging was performed with Image J software (version 1.52a) to quantify the distribution area of cancer cells.

### 4.17. Statistical Analysis

All analyses were performed using GraphPad Prism 8.0 (GraphPad Software, San Diego, CA, USA). All results were presented as mean ± standard deviation (SD) and analyzed by one-way ANOVA. Comparisons between the groups were made using Dunnett’s T-test. The level of statistical significance was set at * *p* < 0.05, ** p <0.01.

## Figures and Tables

**Table 1:** Affinity of pyripyropene O binding to various proteins predicted by molecular docking.

Protein Name	PDB ID	Affinity (kcal/mol)	Estimated Ki	Ligand Efficiency
DR5	2H9G	−8.9	299.41 nM	−0.24
YY1	4C5I	−11.2	6.17 nM	−0.3
SP1	1VA1	−6.9	8.76 μM	−0.19
AP1	1A02	−9.2	180.45 nM	−0.25
TP53	5HOU	−7.4	3.77 μM	−0.20
NF-κB	1SVC	−7.5	3.18 μM	−0.20
CEBP	6DC0	−8.3	824.26 nM	−0.22

**Table 2:** Absorption.

Property	Value	Decision	Comment
Caco-2 Permeability	−5.028	●	Optimal: higher than −5.15 Log unit
MDCK Permeability	2.1 × 10^−5^	●	■ low permeability: <2 × 10^−6^ cm/s ■ medium permeability: 2–20 × 10^−6^ cm/s ■ high passive permeability: >20 × 10^−6^ cm/s
Pgp-inhibitor	0.996	●	■ Category 1: Inhibitor; Category 0: Non inhibitor; ■ The output value is the probability of being Pgp-inhibitor
Pgp-substrate	0.001	●	■ Category 1: substrate; Category 0: Non-substrate; ■ The output value is the probability of being Pgp-substrate
HIA	0.004	●	■ Human Intestinal Absorption ■ Category 1: HIA+ (HIA < 30%); Category 0: HIA−(HIA < 30%); The output value is the probability of being HIA+
F20%	0.51	●	■ 20% Bioavailability ■ Category 1: F_20%_+ (bioavailability < 20%); Category 0: F_20_−(bioavailability ≥ 20%); The output The output value is the probability of being F20%
F30%	0.988	●	■ 30% Bioavailability ■ Category 1: F_30%_+ (bioavailability < 30%); Category 0: F_30%_−(bioavailability ≥ 30%); The output value is the probability of being F30%

Note for Caco-2 Permeability: > −5.15, excellent (green); otherwise, poor (red). Note for MDCK Permeability: >2 × 10^−6^ cm/s, excellent (green); otherwise, poor (red). Note for Pgp-inhibitor, Pgp-substrate, HIA, F_20%_, F_30%_: 0–0.3, excellent (green); 0.3–0.7, medium (yellow); 0.7–1.0(++), poor (red).

**Table 3:** Distribution.

Property	Value	Decision	Comment
PPB	80.78%	●	■ Plasma Protein Binding ■ Optimal: < 90%. Drugs with high protein-bound may have a low therapeutic index.
VD	1.0	●	■ Volume Distribution ■ Optimal: 0.04–20 L/kg
BBB Penetration	0.176	●	■ Blood-Brain Barrier Penetration ■ Category 1: BBB+; Category 0: BBB−; The output value is the probability of being BBB+
Fu	21.33%	●	■ The fraction unbound in plasms ■ Low: <5%; Middle: 5~20%; High: > 20%

Note for PPB: ≤90%, excellent (green). Note for VD: 0.04–20, excellent (green). Note for BBB Penetration: 0–0.3, excellent (green). Note for Fu: ≥5%, excellent (green).

**Table 4:** Metabolism.

Property	Value	Comment
CYP1A2 inhibitor	0.205	■ Category 1: Inhibitor; Category 0: Non-inhibitor; ■ The output value is the probability of being inhibitor.
CYP1A2 substrate	0.288	■ Category 1: Substrate; Category 0: Non-substrate; ■ The output value is the probability of being substrate.
CYP2C19 inhibitor	0.177	■ Category 1: Inhibitor; Category 0: Non-inhibitor; ■ The output value is the probability of being inhibitor.
CYP2C19 substrate	0.553	■ Category 1: Substrate; Category 0: Non-substrate; ■ The output value is the probability of being substrate.
CYP2C9 inhibitor	0.326	■ Category 1: Inhibitor; Category 0: Non-inhibitor; ■ The output value is the probability of being inhibitor.
CYP2C9 substrate	0.157	■ Category 1: Substrate; Category 0: Non-substrate; ■ The output value is the probability of being substrate.
CYP2D6 inhibitor	0.077	■ Category 1: Inhibitor; Category 0: Non-inhibitor; ■ The output value is the probability of being inhibitor.
CYP2D6 substrate	0.147	■ Category 1: Substrate; Category 0: Non-substrate; ■ The output value is the probability of being substrate.
CYP3A4 inhibitor	0.81	■ Category 1: Inhibitor; Category 0: Non-inhibitor; ■ The output value is the probability of being inhibitor.
CYP3A4 substrate	0.478	■ Category 1: Substrate; Category 0: Non-substrate; ■ The output value is the probability of being substrate.

**Table 5:** Excretion.

Property	Value	Decision	Comment
CL	2.193	●	■ Clearance ■ High: >15 mL/min/kg; moderate: 5–15 mL/min/kg; low: <5 mL/min/kg
T_1/2_	0.278	-	■ Category 1: long half-life; Category 0: short half-life; ■ long half-life: >3 h; short half-life: <3 h ■ The output value is the probability of having long half-life.

Note for CL: <5, poor (red).

**Table 6:** PyrO Toxicity Simulation Results.

Property	Value	Decision	Comment
hERG Blockers	0.583	●	■ Category 1: active; Category 0: inactive. ■ The output value is the probability of being active.
H-HT	0.777	●	■ Human Hepatotoxicity ■ Category 1: H-HT positive (+); Category 0: H-HT negative (−). ■ The output value is the probability of being toxic.
DILI	0.822	●	■ Drug Induced Liver Injury. ■ Category 1: drugs with a high risk of DILI; Category 0: drugs with no risk of DILI. The output value is the probability of being toxic.
AMESToxicity	0.011	●	■ Category 1: Ames positive (+); Category 0: Ames negative (−); ■ The output value is the probability of being toxic.
Rat Oral Acute Toxicity	0.104	●	■ Category 0: low-toxicity; Category 1: high-toxicity. ■ The output value is the probability of being highly toxic.
FDAMDD	0.454	●	■ Maximum Recommended Daily Dose ■ Category 1: FDAMDD (+); Category 0: FDAMDD (−) ■ The output value is the probability of being positive.
Skin Sensiti zation	0.566	●	■ Category 1: Sensitizer; Category 0: Non-sensitizer. ■ The output value is the probability of being sensitizer.
Carcinogen city	0.067	●	■ Category 1: carcinogens; Category 0: non-carcinogens. ■ The output value is the probability of being toxic.
Eye Corrosion	0.003	●	■ Category 1: corrosives; Category 0: noncorrosives ■ The output value is the probability of being corrosives.
Eye Irritation	0.009	●	■ Category 1: irritants; Category 0: nonirritants ■ The output value is the probability of being irritants.
Respiratory Toxicity	0.929	●	■ Category 1: respiratory toxicants; Category 0: respiratory nontoxicants ■ The output value is the probability of being toxic.

Note: 0–0.3, excellent (green); 0.3–0.7, medium (yellow); 0.7–1.0 (++), poor (red).

## Data Availability

The original contributions presented in the study are included in the article/Supplementary Material; further inquiries can be directed to the corresponding author.

## Supplementary Materials

The following supporting information can be downloaded at: https://www.mdpi.com/article/10.3390/md23050214/s1. Figure S1: Apoptosis in PC-3 cells treated with PyrO for 72 h; Figure S2: The ^1^H NMR Spectrum of pyripyropene O in DMSO; Figure S3: The ^13^C NMR Spectrum of pyripyropene O in DMSO; Figure S4: The HRESIMS spectrum of pyripyropene O; Figure S5: Expression of YY1 in different cell lines. Reference [58] is cited in the Supplementary Materials.

## Abbreviations

The following abbreviations are used in this manuscript:

ADMET	Absorption, Distribution, Metabolism, Excretion, Toxicity
AMdock	Assisted molecular docking
AR	Androgen receptor
BCA	Bicinchoninic acid
CETSA	Cellular thermal shift assay
CRPC	Castration-resistant prostate cancer
DMSO	Dimethyl sulfoxide
DR5	Death receptor 5
FBS	Fetal bovine serum
KD	Dissociation affinity constant
mCRPC	Metastatic castration resistant prostate cancer
mHSPC	Metastatic hormone-sensitive prostate cancer
MNP	Marine natural products
mRNA	Messenger RNA
MTT	3-(4,5-dimethyl-2-thiazolyl)-2,5-diphenyl-2-H-tetrazolium bromide
PBS	Phosphate buffered saline
PCa	prostate cancer
PDB	Protein data bank
PMSF	Phenylmethanesulfonyl fluoride
PyrO	Pyripyropene O
PVDF	Polyvinylidene fluoride
RIPA	Radio immunoprecipitation assay
RTCA	Real time cellular analysis
YY1	Yin And Yang 1 Protein
