# Supplementary material for: Targeting YY1-DR5 Axis by Pyripyropene O as a Novel Therapeutic Strategy Against Prostate Cancer: Molecular Mechanisms and In Vivo Zebrafish Validation
Source: Mar Drugs. 2025 May 19;23(5):214. doi: 10.3390/md23050214 (PMC12113610; doi:10.3390/md23050214)
Supplement: Supplementary file 1 [file marinedrugs-23-00214-s001.zip › marinedrugs-3616901-supplementary.pdf]

Targeting YY1-DR5 Axis by Pyripyropene O as a Novel Therapeutic Strategy against Prostate Cancer: Molecular mechanisms and in vivo zebrafish validation

Wenxuan Fang [a, b, #], Ying Chen [c, #], Mingyi Nie [a, b], Xuefeng zhou [c], Yonghong Liu [b, c], Huaming Tao [d, *], Bin Yang [c, *], Xueni Wang [a, b, *]

[a] Guangxi Innovation Center of Zhuang Yao Medicine, Institute of Traditional Chinese and Zhuang-Yao Ethnic Medicine, Guangxi University of Chinese Medicine, Nanning 530200, China

[b] Guangxi key laboratory of marine drugs, Institute of marine drugs, Guangxi University of Chinese Medicine, Nanning 530200, China

[c] CAS Key Laboratory of Tropical Marine Bio-resources and Ecology/Guangdong Key Laboratory of Marine Materia Medica, South China Sea Institute of Oceanology, Chinese Academy of Sciences, Guangzhou 510301, China

[d] School of Traditional Chinese Medicine, Southern Medical University, Guangzhou 510515, China

[#] These authors contributed equally to this work.

[*] Corresponding author. E-mail: Huaming Tao, taohm@smu.edu.cn; Bin Yang, yangbin@scsio.ac.cn; Xueni Wang, wangxueni@gxtcmu.edu.cn.

Supplementary Material

1. Figure S1

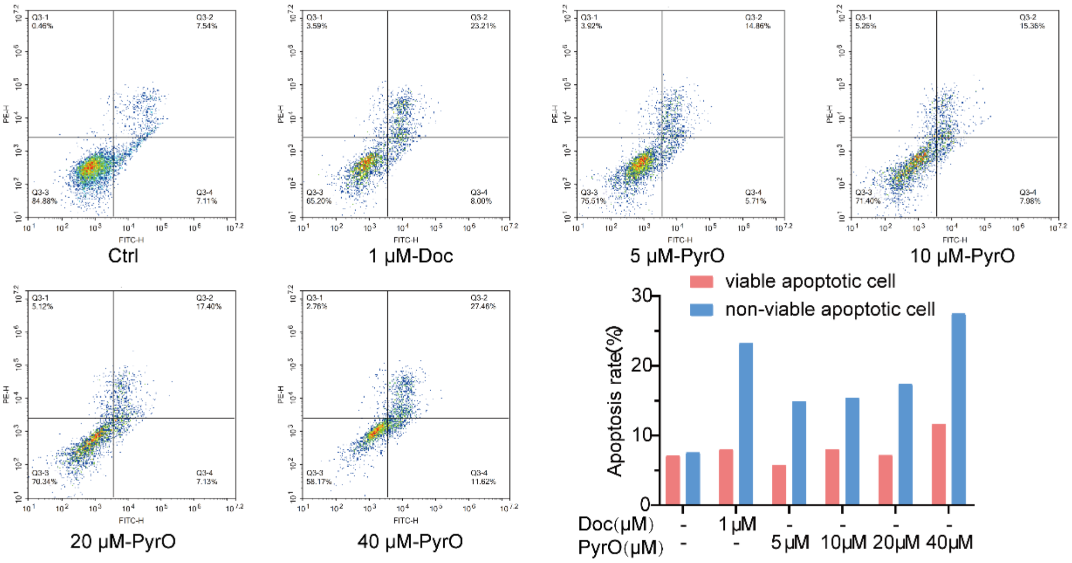

Figure S1 PyrO induces apoptosis in PC-3 cells

This figure illustrates the apoptosis of PC-3 cells after 72 hours of treatment with compound PyrO (5, 10, 20, and 40 μM). The chart counts the ratio of viable apoptotic cells to non-viable apoptotic cells in PC-3 cells treated with different concentrations of PyrO.

2. Figure S2

The ^{1}H NMR spectrum of this compound revealed the presence of five methyl groups. [δ_H 1.24 (s, 3H, H-14), 0.93 (s, 3H, H-12), 0.82 (s, 3H, H-15), 2.01 (s, 3H, 11-CH_3), 1.99 (s, 3H, 1-CH_3)], six methylene groups [δ_H 3.81 (d, *J* = 11.7 Hz, 1H, H-11), 3.69 (d, *J* = 11.7 Hz, 1H, H-11,), 2.37 (m, 1H, H-13), 2.31 (m, 1H, H-13), 2.18 (m, 2H, H-7, H-8), 2.07 (m, 2H, H-3, H-2), 1.19 (m, 1H, H-3), 1.67 (m, 1H, H-2), 1.67 (m, 1H, H-7), 1.43 (m, 1H, H-8)] with one oxygen-linked methylene group and three methine groups [δ_H 4.65 (dd, *J* = 11.4, 5.1 Hz, 1H, H-1), 1.59 (m, 1H, H-5), 1.43 (m, 1H, H-9)], five olefinic protons

[δ_H 9.05 (d, *J* = 2.4 Hz, 1H, H-2’’), 8.66 (dd, *J* = 4.9, 1.6 Hz, 1H, H-6’’), 8.21 (m, 1H, H-4’’), 7.52 (dd, *J* = 8.1, 4.8 Hz, 1H, H-5’’), 6.99 (s, 1H, H-5’)].

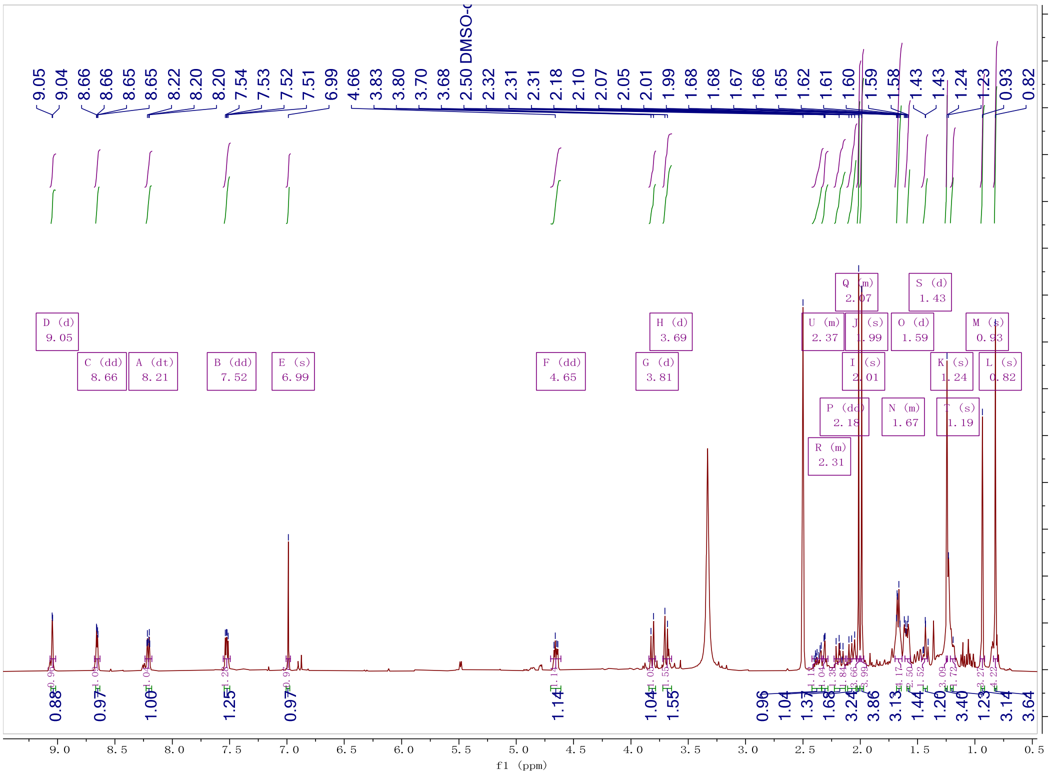

Figure S2. The ^1H NMR Spectrum of pyripyropene O in DMSO

^1H NMR (500 MHz, DMSO-d_6) δ 9.05 (d, *J* = 2.4 Hz, 1H, H-2’’), 8.66 (dd, *J* = 4.9, 1.6 Hz, 1H, H-6’’), 8.21 (m, 1H, H-4’’), 7.52 (dd, *J* = 8.1, 4.8 Hz, 1H, H-5’’), 6.99 (s, 1H, H-5’), 4.65 (dd, *J* = 11.4, 5.1 Hz, 1H, H-1), 3.81 (d, *J* = 11.7 Hz, 1H, H-11), 3.69 (d, *J* = 11.7 Hz, 1H, H-11,), 2.37 (m, 1H, H-13), 2.31 (m, 1H, H-13), 2.18 (m, 1H, H-7), 2.07 (m, 2H, H-3, H-2), 2.01 (s, 3H, 11-CH_3), 1.99 (s, 3H, 1-CH_3), 1.67 (m, 2H, H-7, H-8), 1.59 (m, 1H, H-5), 1.43 (m, 1H, H-9), 1.24 (s, 3H, H-14), 1.19 (m, 1H, H-3), 0.93 (s, 3H, H-12), 0.82 (s, 3H, H-15).

3. Figure S3

The ^{13}C NMR spectrum of the compound exhibited 29 distinct carbon signals corresponding to five methyl groups, six methylene groups, three methine groups, four

olefinic carbons, five carbons assigned to a pyridine ring system, two carbonyl groups, and three quaternary carbons. Comparative analysis of the NMR data with literature values demonstrated strong consistency with those of pyripyropene O, thereby conclusively establishing the identity of compound as pyripyropene O.

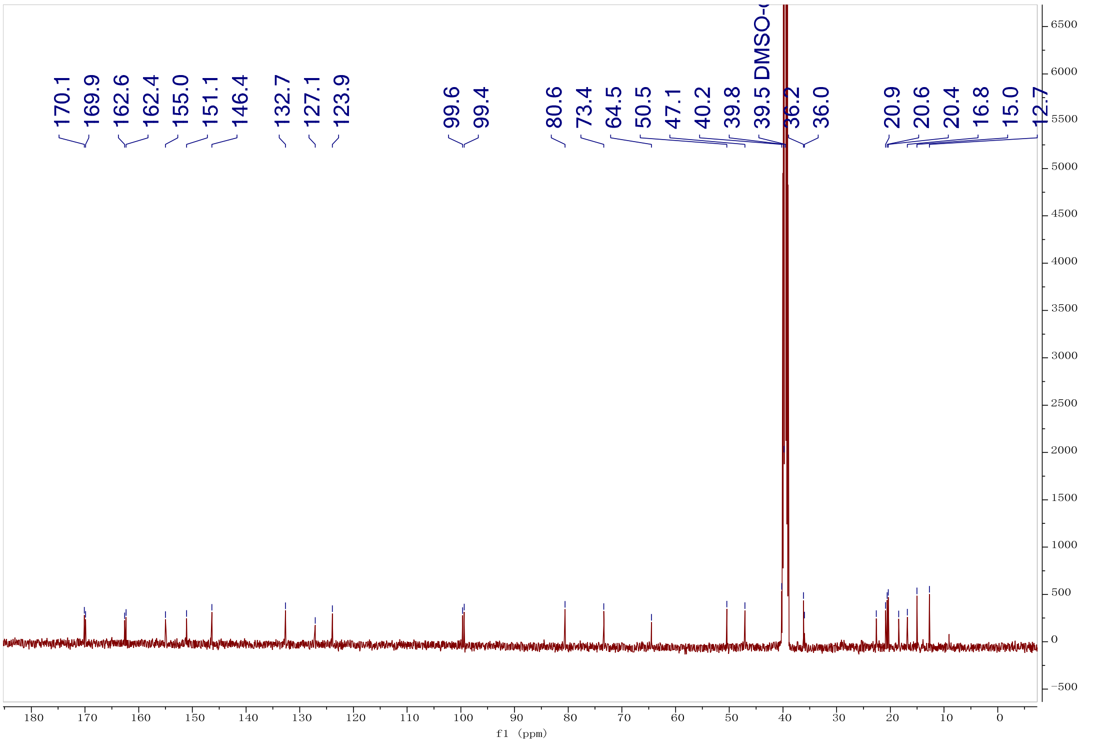

Figure S3. The ^{13}C NMR Spectrum of pyripyropene O in DMSO

^{13}C NMR (126 MHz, DMSO) δ 170.1 (C, 11-CO), 169.9 (C, 1-CO), 162.6 (C, C-2'), 162.4 (C, C-4'), 155.0 (C, C-6'), 151.1 (CH, C-6''), 146.4 (CH, C-2''), 132.7 (CH, C-4''), 127.1 (C, C-3''), 123.9 (CH, C-5''), 99.6 (C, C-3'), 99.4 (CH, C-5'), 80.6 (C, C-6), 73.4 (CH, C-1), 64.5 (CH_2, C-11), 50.5 (CH, C-5), 47.1 (CH, C-9), 40.2 (C, C-10), 39.8 (CH2, C-7), 36.2 (CH_2, C-3), 36.0 (C, C-4), 22.6 (CH_2, C-2), 20.9 (CH_3, 1-CH_3), 20.6 (CH_3, 11-CH_3) 20.4 (CH_3, C-14), 18.4 (CH_2, C-8), 16.8 (CH_2, C-13), 15.0 (CH_3, C-12), 12.7 (CH_3, C-15).

4. Figure S4

Pyripyropene O was obtained as a pale-yellow powder with a molecular formular of

$C_{29}H_{35}NO_7$, determined by HRESIMS analysis (*m/z* 510.2486 $[M+H]^+$).

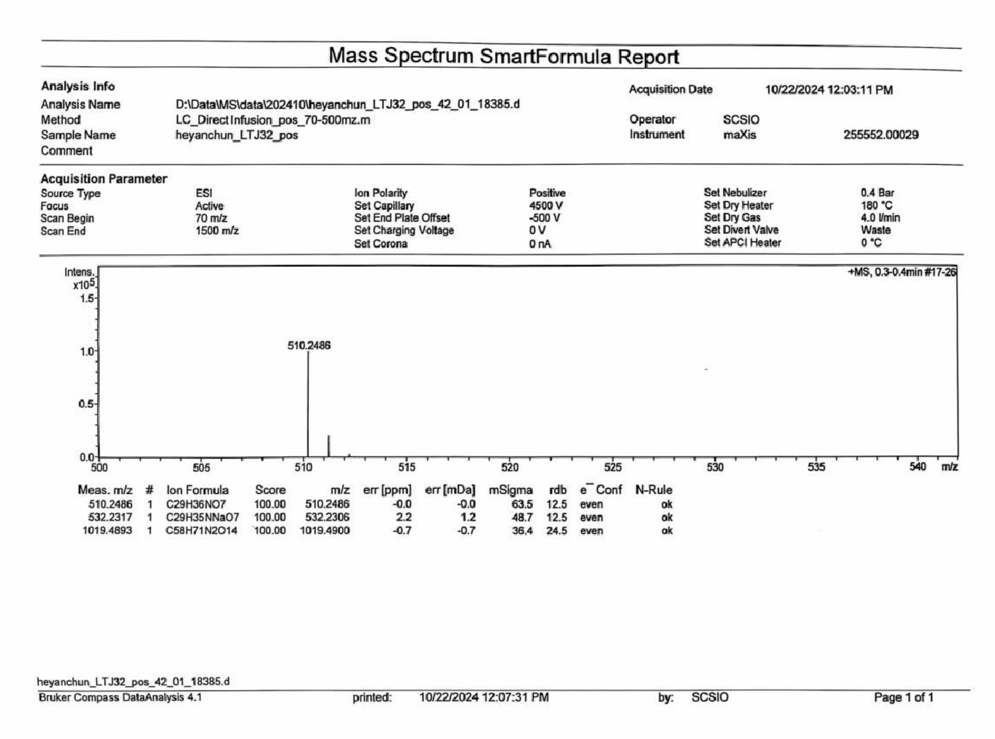

Figure S4 The HRESIMS spectrum of pyripyropene O

5. Figure S5

The gene expression matrix for prostate cancer cell lines was obtained from the CCLE dataset (https://depmap.org/portal/data_page/?tab=allData). Statistical analysis was conducted using R software, version v4.0.3. Results were considered statistically significant when the p-value was less than 0.05.

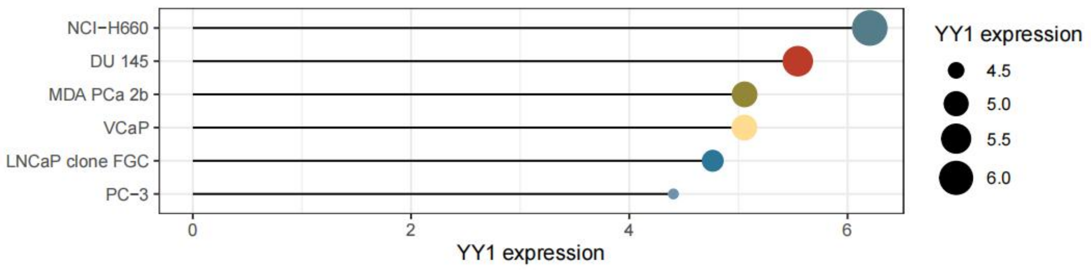

Figure S5 Expression of YY1 in different cell lines

The graph illustrates the distribution of gene expression across different cell lines. The horizontal axis represents the expression level of the gene, while the vertical axis lists the different cell lines. The size of the dots in the graph reflects the level of gene

expression.

References

Ghandi M, Huang FW, Jané-Valbuena J, Kryukov GV, Lo CC, McDonald ER 3rd, Barretina J, Gelfand ET, Bielski CM, Li H, Hu K, Andreev-Drakhlin AY, Kim J, Hess JM, Haas BJ, Aguet F, Weir BA, Rothberg MV, Paolella BR, Lawrence MS, Akbani R, Lu Y, Tiv HL, Gokhale PC, de Weck A, Mansour AA, Oh C, Shih J, Hadi K, Rosen Y, Bistline J, Venkatesan K, Reddy A, Sonkin D, Liu M, Lehar J, Korn JM, Porter DA, Jones MD, Golji J, Caponigro G, Taylor JE, Dunning CM, Creech AL, Warren AC, McFarland JM, Zamanighomi M, Kauffmann A, Stransky N, Imielinski M, Maruvka YE, Cherniack AD, Tsherniak A, Vazquez F, Jaffe JD, Lane AA, Weinstock DM, Johannessen CM, Morrissey MP, Stegmeier F, Schlegel R, Hahn WC, Getz G, Mills GB, Boehm JS, Golub TR, Garraway LA, Sellers WR. Next-generation characterization of the Cancer Cell Line Encyclopedia. Nature. 2019 May;569(7757):503-508.
